# Single-Dose Anti-CD138 Radioimmunotherapy: Bismuth-213 is More Efficient than Lutetium-177 for Treatment of Multiple Myeloma in a Preclinical Model

**DOI:** 10.3389/fmed.2015.00076

**Published:** 2015-11-04

**Authors:** Nolwenn Fichou, Sébastien Gouard, Catherine Maurel, Jacques Barbet, Ludovic Ferrer, Alfred Morgenstern, Frank Bruchertseifer, Alain Faivre-Chauvet, Edith Bigot-Corbel, François Davodeau, Joëlle Gaschet, Michel Chérel

**Affiliations:** ^1^Centre Régional de Recherche en Cancérologie Nantes/Angers (CRCNA) – UMR 892 INSERM, Université de Nantes, Nantes, France; ^2^CNRS 6299, Université de Nantes, Nantes, France; ^3^Université de Nantes, Nantes, France; ^4^Institut de Cancérologie de l’Ouest, Saint-Herblain, France; ^5^Institute for Transuranium Elements, Karlsruhe, Germany; ^6^Nuclear Medicine Department, CHU Nantes, Nantes, France

**Keywords:** lutetium-177, bismuth-213, multiple myeloma, CD138, radioimmunotherapy

## Abstract

**Objectives:**

Radioimmunotherapy (RIT) has emerged as a potential treatment option for multiple myeloma (MM). In humans, a dosimetry study recently showed the relevance of RIT using an antibody targeting the CD138 antigen. The therapeutic efficacy of RIT using an anti-CD138 antibody coupled to ^213^Bi, an α-emitter, was also demonstrated in a preclinical MM model. Since then, RIT with β-emitters has shown efficacy in treating hematologic cancer. In this paper, we investigate the therapeutic efficacy of RIT in the 5T33 murine MM model using a new anti-CD138 monoclonal antibody labeled either with ^213^Bi for α-RIT or ^177^Lu for β-RIT.

**Methods:**

A new monoclonal anti-CD138 antibody, 9E7.4, was generated by immunizing a rat with a murine CD138-derived peptide. Antibody specificity was validated by flow cytometry, biodistribution, and α-RIT studies. Then, a β-RIT dose-escalation assay with the ^177^Lu-radiolabeled 9E7.4 mAb was performed in KalwRij C57/BL6 mice 10 days after i.v. engraftment with 5T33 MM cells. Animal survival and toxicological parameters were assessed to define the optimal activity.

**Results:**

α-RIT performed with 3.7 MBq of ^213^Bi-labeled 9E7.4 anti-CD138 mAb increased median survival to 80 days compared to 37 days for the untreated control and effected cure in 45% of animals. β-RIT performed with 18.5 MBq of ^177^Lu-labeled 9E7.4 mAb was well tolerated and significantly increased mouse survival (54 vs. 37 days in the control group); however, no mice were cured with this treatment.

**Conclusion:**

This study revealed the advantages of α-RIT in the treatment of MM in a preclinical model where β-RIT shows almost no efficacy.

## Introduction

Multiple myeloma (MM) represents 1% of all cases of cancer and 10% of hematological malignancies (1). The physiopathology of MM consists of an uncontrolled proliferation of a monoclonal plasma cell in the bone marrow, resulting in the abnormal secretion of a monoclonal immunoglobulin, known as paraprotein, which can be detected in patients’ serum or urine. Common features of MM include organ damage due to plasma cell proliferation – also known as CRAB (hypercalcemia, renal failure, anemia, and bone diseases) – at least one focal lesion, and a ratio of involved:uninvolved circulating light chain higher than 100 (2, 3). Depending on the patient’s age, standard clinical care comprises high-dose therapy, which may be combined with autologous stem cell transplantation (4). Although new drugs such as bortezomib and lenalidomide, and new indications, such as for thalidomide, have significantly improved the overall survival rate for patients, MM remains an incurable disease. Therefore, treatments must be constantly improved and new drugs or new strategies continuously assessed.

In the past two decades, radioimmunotherapy (RIT) has emerged as a novel strategy in the anti-cancer arsenal. This therapeutic approach consists in specifically targeting tumor cells using an antibody coupled with a radionuclide. The radionuclide can then kill tumor cells by direct cytotoxicity as well as through a bystander effect (5–9). Among the radionuclides which have been tested in clinical trials, there are a few β^–^ emitters, such as ^131^I, ^90^Y, ^177^Lu, and a number of α-emitters such as ^213^Bi, ^211^At, or ^225^Ra (10–15). The efficacy of RIT depends on the choice of radionuclide, which is determined by the vector and the nature of the cancer (16). Indeed, the characteristics of α- and β-ionizing radiation differ, with α-particles having a much higher linear energy transfer than β^−^ emitters. Their range of penetration into tissues also differs: 50–100 μm for α-particles vs. 2–10 mm for β^−^ particles. Based on these characteristics, α-particles appear better suited to treating disseminated cancer or micrometastases. The proof of concept of α-RIT in humans was provided by a clinical trial using an anti-CD33 mAb coupled to ^213^Bi as a treatment for myeloid leukemia (17). In this study, which was conducted in patients with large tumor burdens, no significant extramedullary toxicity was observed, nor was any complete response elicited supporting the hypothesis that α-RIT is more appropriate for treating small tumor volumes, residual disease, and micrometastases. Several clinical trials using β-RIT have also shown promising results with hematologic diseases, such as non-Hodgkin’s lymphomas (NHL) (18, 19) and with solid tumors, such as metastatic prostate cancer (20, 21). ^177^Lu appears to be an excellent candidate for RIT because of its short range of action (2 mm in tissues). This range is similar to that of ^131^I (2.9 mm), but shorter than that of ^90^Y (11 mm). This characteristic may reduce non-specific radiation damage to surrounding healthy tissues. In addition, its low ɣ-emission (208 keV) is an advantage in terms of radioprotection.

Our group has recently proven RIT to be effective in treating MM in humans (22) and α-RIT to be effective in an immunocompetent preclinical MM model (23). Both studies were performed with antibodies targeting the CD138 antigen, which is expressed by more than 95% of MM cells (24). CD138, or syndecan-1, is a member of the syndecan family of heparan sulfate proteoglycans; it is expressed by epithelial cells, precursor B cells, and normal plasma cells. It is also expressed at high levels in MM tumors (24–26) and is a key regulator in the disease (27). Finally, high levels of CD138 in patient serum are associated with poor prognosis in MM disease progression (28–30).

Based on our study in MM patients with ^131^I-CD138 mAb (22), and the promising results obtained in treatment of NHL patients with β-RIT using an anti-CD20 mAb labeled with ^90^Y and ^177^Lu (18, 19), we decided to investigate the therapeutic efficacy of β-RIT in a preclinical model of MM using an anti-CD138 mAb coupled to ^177^Lu. We first generated and validated a new murine anti-CD138 antibody (9E7.4 mAb) and confirmed that it could effectively treat MM tumors using α-RIT. Then, we studied the tissue distribution of ^177^Lu-9E7.4 mAb and went on to perform dose-escalation and toxicity studies. Finally, we administered ^177^Lu-9E7.4 at different times during disease progression.

## Materials and Methods

### Reagents, Cell Lines, and Mice

The bifunctional chelators S-2-(4-Isothiocyanatobenzyl)-1,4,7,10-tetraazacyclododecane tetraacetic acid (p-SCN-Bn-DOTA) and 2-(4-isothiocyanato-benzyl)-cyclohexyl-diethyletriaminepenta-acetic (SCN-CHX-A″-DTPA) were purchased from Macrocyclics (USA). ^177^LuCl_3_ was purchased from Perkin Elmer (France) in 0.05 N HCl and ^225^Ac/^213^Bi generator was obtained from the Institute for Transuranium Elements (Karlsruhe, Germany). All buffers were prepared in H_2_O milliQ (0.2 μm; 3 ppb; 18 MΩ) and Trace SELECT chemicals, when available. Rat IgG2a,κ anti-mouse CD138 antibody (clone 281-2) (ref.553712) was purchased from BD Pharmingen (France).

The 5T33 murine MM cell line was kindly provided by Dr. Harvey Turner (Department of Nuclear Medicine, Fremantle Hospital, Western Australia) with permission from Dr. J. Radl (TNO Institute, Leiden, The Netherlands) (31, 32). The SP2/o-Ag14 myeloma cell line (ATCC^®^ CRL-1581™) and the CTLL-2 T lymphocyte cell line (ATCC^®^ TIB-214™) were purchased from ATCC. All cell lines were cultured in RPMI 1640 medium (Life Technologies, France) containing 2 mM l-glutamine, 100 U/mL penicillin/100 μg/mL streptomycin and 10% heat-inactivated fetal calf serum (Life Technologies, France). Cells were incubated at 37°C, 5% CO_2_ in a humidity-saturated incubator.

Female C57BL/KalwRij mice were purchased from Harlan CPB and housed under conventional conditions at the UTE animal facility (SFR François Bonamy, IRS-UN, University of Nantes, license number: B-44-278). Experiments were approved by the local veterinary committee (License No. CEEA.2013.2). Mice were 8–12 weeks old at the time of experiments.

### Anti-CD138 9E7.4 mAb Production and Characterization

The 9E7.4 mAb was produced by immunization of a rat with a 40-amino-acid peptide (GeneCust, Luxembourg) derived from the murine CD138 protein (aa 90–130) (GenBank: CAA80254.1). The isotype of 9E7.4 immunoglobulin was determined using a Rat Isotyping Kit (RMT1, AbD serotec, UK) according to the manufacturer’s instructions.

The specificity and affinity of 9E7.4 mAb for CD138 were determined by flow cytometry (FACS Calibur™, BD Biosciences). Staining was detected indirectly with a secondary antibody, anti-rat IgG/PE (Jackson-ImmunoResearch Laboratories).

### Anti-CD138 mAb Conjugation

A reaction solution containing a 20M excess of p-SCN-Bn-DOTA or SCN-CHX-A″-DTPA over the 9E7.4 mAb in 0.3M carbonate buffer pH 8.7 was incubated overnight (16 h) at room temperature (RT). Free chelator was eliminated and the conjugation buffer was exchanged for 0.25M NH_4_OAc, pH 7 (DOTA-9E7.4 mAb) or for PBS (CHX-A″-DTPA-9E7.4 mAb) by several centrifugal filtration steps using a 30-kDa cutoff Centricon concentrator (Amicon, Millipore). Integrity of the immunoconjugate was verified by size-exclusion HPLC. The conjugated 9E7.4 mAb was filtered through a 0.2-μm filter before storage at 4°C. The number of chelators per antibody was estimated at between 1 and 4 for p-SCN-Bn-DOTA and 2 for SCN-CHX-A″-DTPA by an ^111^In dilution assay (23) and/or mass spectrometry.

### Anti-CD138 mAb Radiolabeling

^177^Lu-chloride (370 MBq) was added to a solution containing DOTA-9E7.4 mAb, prepared in 0.25M NH_4_OAc pH 7 and 0.05M ascorbic acid buffer pH 5.5, and was adjusted to pH 5.5 with 0.5M NH_4_OAc. The reaction mixture was incubated at 42 ± 1°C for 3 h. Complexation was then stopped by adding a 10M excess of EDTA and mixing for 15 min at RT. Labeling of anti-CD138 9E7.4 mAb with ^213^Bi was performed as described in Chérel et al. (23). Radioiodination of anti-CD138 antibodies was performed as described in Fraker and Speck (33). All radiolabeled immunoconjugates were purified by gel filtration on a Sephadex G-25 PD-10 desalting column (GE Healthcare Life Science, France) and eluted in 0.9% NaCl. The radiochemical purity was verified by instant thin layer chromatography (ITLC-SG) as described in Koppe et al. (34), using citrate buffer (0.1M pH 4.5) as the mobile phase. For all experiments, purities of the ^177^Lu and ^213^Bi immunoconjugates were >98%, and purity of the ^125^I immunoconjugate was >95%. The immunoreactive fraction of the radiolabeled conjugates was determined using CD138-peptide-coated magnetic beads. In all cases, the immunoreactive fraction was estimated at ≥80%.

### Biodistribution

To determine the biodistribution of anti-mouse CD138 mAb, 3 μg of ^125^I immunoconjugate were administered intravenously (i.v.) to tumor-free C57BL/KalwRij (*n* = 3 in each group). Mice were sacrificed 5 min, 4, and 24 h later. To study the tissue distribution of ^177^Lu-9E7.4 mAb, 3 μg were i.v. injected into tumor-free C57BL/KalwRij (*n* = 3 in each group). Mice were sacrificed 5 min, 2, 8, 24, 48, 96, or 168 h later. For all experiments, tissues were collected and radioactivity was counted using a ɣ-counter (Wizard 1480, PerkinElmer, France). To account for physical decay of radioactivity, a triplicate standard dose was counted alongside the samples, and results were expressed as a percentage of injected dose per gram of tissue.

### Dose-Escalation and Therapeutic Studies

Mice were engrafted with 1 × 10^6^ 5T33 MM cells. α-RIT was then performed on day 10 and β-RIT on day 10, 15, or 20. Animal survival was monitored over time. Mice were sacrificed when paralysis was detected and/or their weight dropped by 20%. In some cases, tumor masses were detected. For α-RIT, a single dose of 3.7 MBq ^213^Bi-9E7.4 mAb (*n* = 20) was injected i.v., in line with the previously described protocol (23). For β-RIT, a dose-escalation study was performed with i.v. injection of 3.7 (*n* = 14), 7.4 (*n* = 5), 11.1 (*n* = 5), 14.8 (*n* = 5), 18.5 (*n* = 15), 27.75 (*n* = 11), or 37 MBq (*n* = 7) ^177^Lu-9E7.4 mAb at a specific activity of 1480 ± 74 MBq/mg of antibody.

### Toxicity Study

To assess hematologic toxicity, blood was collected from the ­retro-orbital sinus for leukocyte, erythrocyte, and thrombocyte counting on an automated hematology analyzer (Melet-Schloesing Laboratories, France). Plasma was isolated from blood samples, pooled, and then used to monitor bone marrow, liver, and kidney toxicity based on the following biomarkers: Flt3-L – measured at different time points using DuoSet ELISA kits (DY427, R&D System, France), according to the supplier’s instructions – ASAT, ALAT, urea, and creatinin – concentrations of these markers were monitored using a Roche Cobas Analyzer (Roche, Meylan, France) with the appropriate reagents (Roche, Meylan, France).

### Statistical Analyses

Data are presented as mean ± SEM. Statistical significance was determined using the Mann–Whitney test. Animal survival data were analyzed using Kaplan–Meier survival curves combined with the log-rank test. All statistical analyses were performed using GraphPad Prism^®^ software (GraphPad Software).

## Results

### Characterization and Validation of the 9E7.4 Antibody

**Figure 1:**
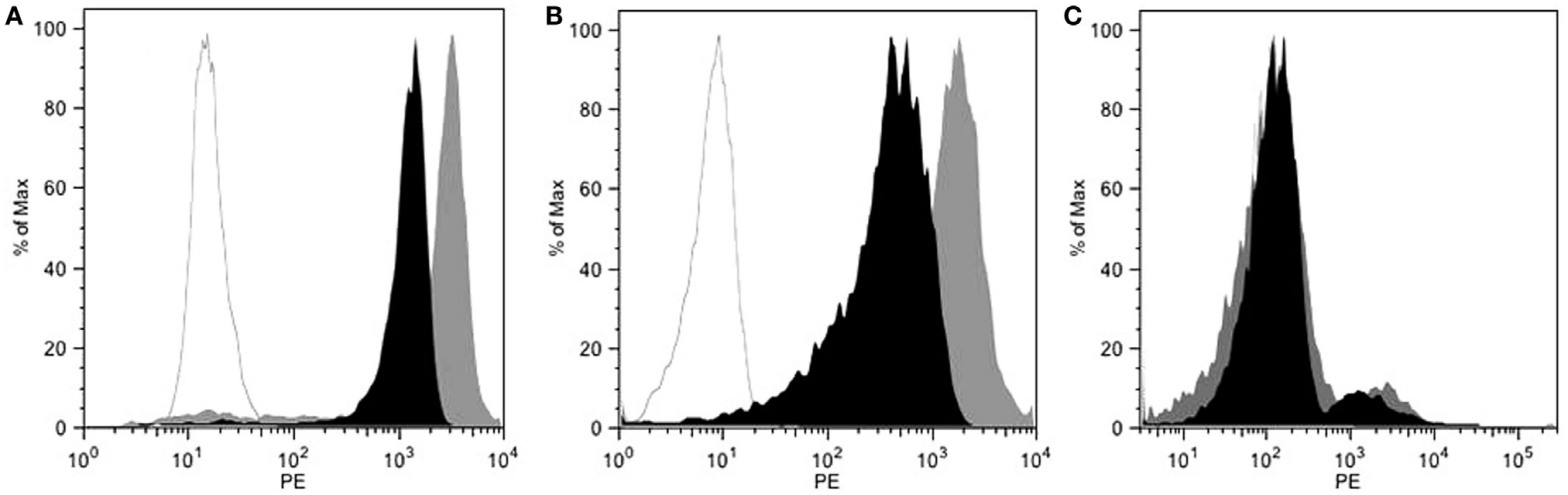
**Flow cytometry characterization of 9E7.4 mAb specificity**. Staining with 9E7.4 mAb (black histogram), 281-2 mAb (gray histogram), or a rat IgG2a,κ isotype control (white histogram) revealed by a PE-conjugated anti-rat IgG secondary antibody performed on **(A)** CD138^+^ 5T33 cell line, **(B)** CD138^+^ SP2/o cell line, and **(C)** CD138^−^ CTLL2 cell line. Flow cytometry was performed on a BD FACS Calibur™ Flow Cytometry System.

**Figure 2:**
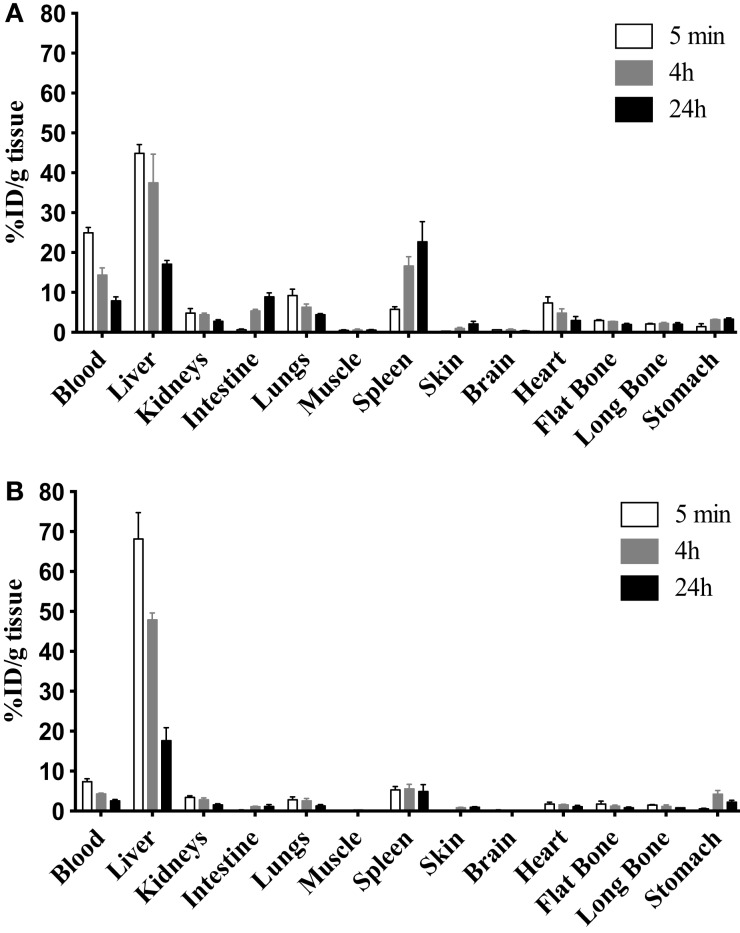
**Biodistribution of the two anti-mouse CD138 mAbs**. To complete 9E7.4 mAb characterization, biodistribution studies were performed in tumor-free C57BL/KalwRij mice by i.v. injection of ^125^I-radiolabeled **(A)** 9E7.4 mAb or **(B)** 281-2 mAb. Tissues were collected at 5 min, 4, and 24 h after injection. Results are presented as the percentage of the injected dose normalized for tissue weight (% ID/g tissue). Values are given as mean ± SD (*n* = 3 mice per time point).

The 9E7.4 mAb was produced by immunizing a rat with a 40-aa peptide derived from the extracellular domain of murine CD138. The isotype of this antibody was determined to be IgG2a,κ, and its binding specificity was initially validated by ELISA using the 40-aa peptide (data not shown). This specificity was confirmed by flow cytometry on different cell lines and compared to that of 281-2 mAb, a commercial anti-mouse CD138 mAb, which we had previously used (23) (Figure 1). The two anti-CD138 mAbs could bind to CD138-expressing MM cell lines 5T33 and SP2/o, whereas no binding to the negative control cell line was observed in either case. An *in vitro* competition assay indicated that these two anti-CD138 mAbs bind to different epitopes on CD138 (data not shown). The binding affinity for mAbs 281-2 and 9E7.4 on 5T33 MM cells was comparable: 1 × 10^−10^ and 2.3 × 10^−10^M, respectively (data not shown). However, maximal binding of the 281-2 mAb was at least twofold more intense than 9E7.4 mAb binding (Figure 1). This difference in maximal binding led us to investigate the *in vivo* behavior of the 9E7.4 anti-CD138 mAb based on its tissue distribution. The two mAbs were ^125^I-radioiodinated before injection into tumor-free C57BL/KalwRij mice, animals were sacrificed at 5 min, 4, and 24 h and the percentage injected dose per gram of tissue (% ID/g tissue) was calculated (Figure 2). These results showed the 9E7.4 mAb and the 281-2 mAb to distribute differently in blood, liver, and spleen. Five minutes after administration, 25 ± 1.3% ID/g tissue for 9E7.4 mAb was detected in the blood compared to 7.4 ± 0.8% ID/g tissue for 281-2 mAb. ^125^I-281-2 mAb was taken up at a higher level in the liver compared to the ^125^I-9E7.4 mAb, which could explain this difference. Liver uptake might be in part related to CD138 expression by mouse hepatocytes as the liver is a hematopoietic organ in mouse (35, 36). Differences were also noted for spleen uptake, with 9E7.4 mAb uptake increasing over time, whereas 281-2 mAb uptake remained stable. This difference may be related to the lower liver uptake, and the higher level of circulating 9E7.4 mAb molecules. Consequently, more molecules could reach the spleen and bind to the CD138^+^ plasma cells present in this secondary lymphoid organ (37). Beyond blood, liver, and spleen, the overall tissue distribution was quite comparable for the two mAbs, with a low uptake in other healthy organs.

**Figure 3:**
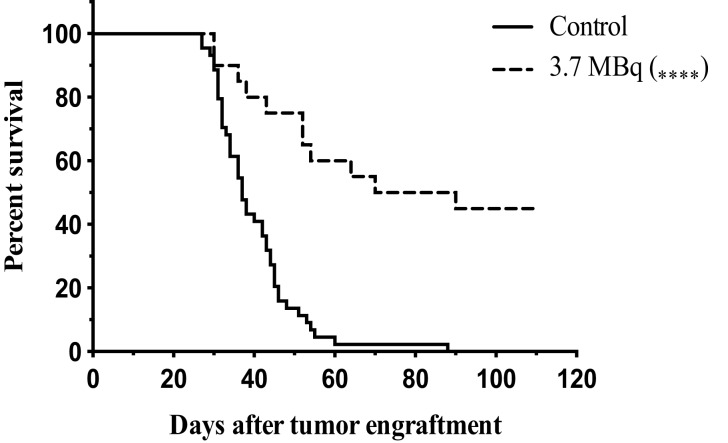
**Survival curves after α-RIT with ^213^Bi-9E7.4 mAb**. At day 0, all mice were engrafted with 1 × 10^6^ of 5T33 cells by i.v. injection. α-RIT was performed on day 10 by i.v. administration of 3.7 MBq ^213^Bi-9E7.4 mAb (dotted line; *n* = 20). The control group (solid line; *n* = 44) received NaCl. *p* values were determined using the log-rank test and found to be highly significant, ****p < 0.0001 (3.7 MBq vs. control).

To further endorse the specificity and relevance of the 9E7.4 mAb, α-RIT using ^213^Bi was performed and the results compared with those from our previous study using the 281-2 mAb (23). Applying the same protocol as described in Chérel et al. (23), 1 × 10^6^ 5T33 MM cells were engrafted in mice, and α-RIT was performed by injecting 3.7 MBq of ^213^Bi-radiolabeled 9E7.4 mAb 10 days later. Survival results are presented in Figure 3 and show that α-RIT significantly increased median survival (80 days) compared to the control group (37 days). Moreover, no mice survived in the control group, whereas 45% of the mice treated by α-RIT were still alive 110 days after tumor engraftment. All these data validated the use of 9E7.4 mAb in therapy.

### Biodistribution of ^177^Lu-9E7.4 mAb

**Figure 4:**
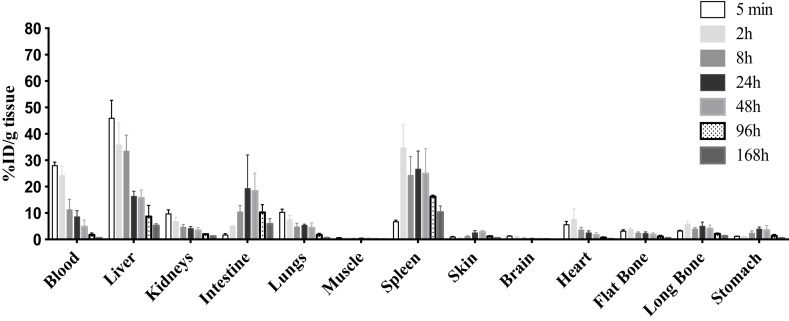
**Biodistribution of ^177^Lu-9E7.4 mAb**. The 9E7.4 mAb radiolabeled with ^177^Lu was injected i.v. into tumor-free C57BL/KalwRij mice (*n* = 3). Tissues were collected 5 min, 2, 8, 24, 48, 96, and 168 h after injection. Results are presented as the percentage of the injected dose normalized for tissue weight (% ID/g tissue).

The tissue distribution of 9E7.4 mAb labeled with ^177^Lu was also studied in tumor-free C57Bl/KalwRij. Results are presented at different time points after injection in Figure 4. Five minutes after injection, 27.9 ± 1.4% ID/g tissue of antibody was detected in blood. As with ^125^I-9E7.4 mAb, a relatively high liver uptake was observed (45.9 ± 6.9% ID/g tissue). This level of uptake was due to specific binding, as confirmed by comparison with a ^177^Lu-isotype control (data not shown). Uptake of ^177^Lu-9E7.4 mAb was also high in the spleen (27.6 ± 8.2% ID/g tissue between 2 and 48 h) and in the intestines (18.8 ± 9.1% ID/g tissue at 24 and 48 h) (Figure 4). Finally, accumulation of the ^177^Lu-9E7.4 mAb in other healthy organs was similar to what has been described with other radioimmunoconjugates (38).

### Dose-Escalation Study with ^177^Lu-9E7.4 mAb

**Figure 5:**
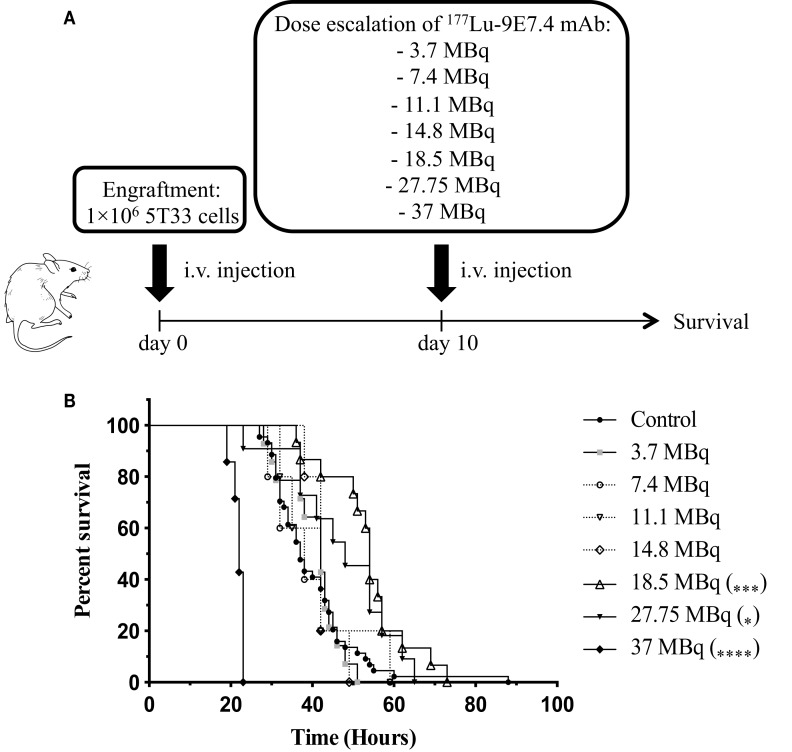
**β-RIT dose-escalation in mice developing MM**. **(A)** Mice were engrafted with 1 × 10^6^ 5T33 cells on day 0. On day 10, “treated” mice received various activities of ^177^Lu-9E7.4 mAb i.v.; “control” mice (*n* = 44) received NaCl. **(B)** Kaplan–Meier survival curves after β-RIT with 3.7 (*n* = 14), 7.4 (*n* = 5), 11.1 (*n* = 5), 14.8 (*n* = 5), 18.5 (*n* = 15), 27.75 (*n* = 11), and 37 MBq (*n* = 7) of ^177^Lu-9E7.4 mAb. ****p < 0.0001 (37 MBq vs. control), ***p = 0.0007 (18.5 MBq vs. control), and *p = 0.0367 (27.75 MBq vs. control), differences between treated and control groups were not statistically significant for other doses. *p* Values were determined by the log-rank test.

Next, we tested the efficacy of β-RIT using the ^177^Lu-radiolabeled 9E7.4 mAb in the 5T33 MM mouse model. As above, mice were engrafted with 1 × 10^6^ 5T33 MM cells 10 days before injecting seven different activities of ^177^Lu-9E7.4 mAb ranging from 3.7 to 37 MBq (Figure 5A). No statistical difference in median survival compared to the control group (37 days) was observed in any group receiving an activity below 18.5 MBq, indicating that below 18.5 MBq, β-RIT had no therapeutic efficacy (Figure 5B). By contrast, survival was significantly increased compared to the control group in mice injected with 18.5 MBq, reaching 54 days. Nevertheless, even with this level of activity, no long-term survival was observed. Interestingly, the group which received 27.75 MBq exhibited a median survival of 48 days, which, although slightly higher than the control group (37 days), is lower than that obtained with 18.5 MBq. Once again, none of the mice survived. This effect was even more dramatic in the 37 MBq group, where median survival was reduced to 22 days – considerably less than the control group. In this group, no sign of paralysis was detected, but all mice had to be sacrificed due to excessive weight loss (Figure S1 in Supplementary Material) suggestive of toxicity rather than MM disease.

Together, these results indicate that 18.5 MBq of ^177^Lu-9E7.4 mAb provides optimal activity in this murine MM model, increasing median survival. However, this activity is not sufficient to cure MM.

### Toxicity Study

**Figure 6:**
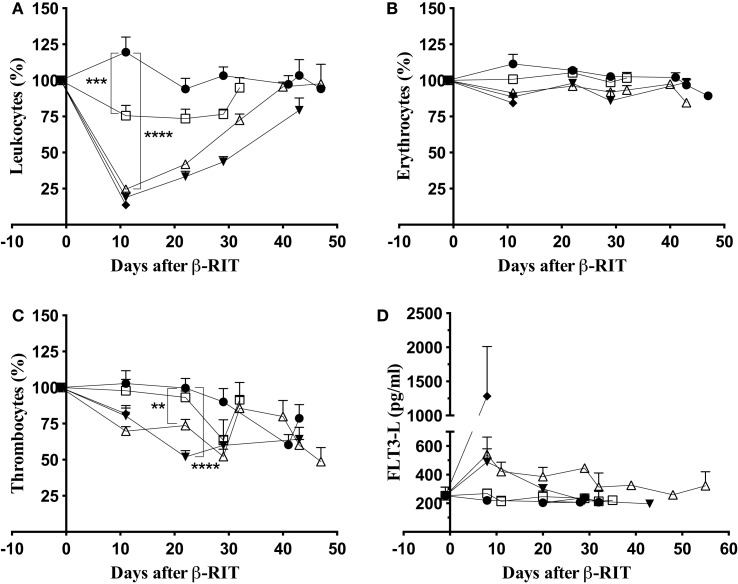
**Hematologic and bone marrow toxicity after β-RIT**. Mice, engrafted with 1 × 10^6^ 5T33cells, were treated with various ^177^Lu-9E7.4 mAb activities at day 10: 3.7 MBq (□), 18.5 MBq (△), 27.75 MBq (▼), 37 MBq (♦), and control (●). **(A)** Leukocyte, **(B)** erythrocyte, and **(C)** thrombocyte counts were monitored using an automated hematology analyzer. Data represent three independent experiments with *n* = 8 (37 MBq), 11 (27.75 MBq), 14 (3.7 MBq), 15 (18.5 MBq), and 20 (control). Results are presented as mean ± SEM. **p = 0.039, ***p = 0.0003, ****p < 0.0001, and *p* values were determined using the Mann–Whitney test. **(D)** FLT3-L concentration in plasma was determined by ELISA. Data represent the mean ± SD for three independent experiments on pooled plasma.

To determine whether the limited efficacy of β-RIT was due to toxicity, we first assessed hematologic toxicity. To do this, leukocyte, erythrocyte, and thrombocyte counts were monitored in the blood at different time points after initiating treatment (Figure 6). Results were normalized against the initial cell counts – before engraftment – and are expressed as percentages. Eleven days after β-RIT with 3.7 MBq, the leukocyte count was reduced by 24.5%; levels returned to normal at day 32 after initiation of the treatment (Figure 6A). With the 18.5 and 27.75 MBq activities, a dramatic nadir was reached with a decrease in leukocyte count of 75.5 and 81.1%, respectively (Figure 6A). A return to normal levels was only achieved with the 18.5 MBq activity 40 days after treatment, while in the 27.75 MBq group, leukocyte counts remained extremely low until the end of the study. Figure 6B shows that no major variations in erythrocyte counts were noted at any activity. By contrast, thrombocytes were also reduced by 26.3% for the 18.5 MBq group and 48.1% for the 27.75 MBq group (Figure 6C). These reductions occurred later than the drop in leukocyte counts at day 22 after β-RIT. Together, these results indicate that of the cell populations examined (1), leukocytes appear to be the most sensitive to ^177^Lu; (2) thrombocytes are also sensitive but to a lesser extent; (3) β-ionizing radiation from ^177^Lu does not directly affect erythrocytes. The hematologic toxicity observed at 18.5 and 27.75 MBq appears significant but reversible, or almost reversible, and might not be a major contributing factor to the mortality observed in these groups of animals.

We next studied non-hematologic toxicity in the bone marrow by monitoring the Flt3-L concentration in plasma (39) at different time points after β-RIT (Figure 6D). In control mice, the Flt3-L concentration was around 250 pg/mL at all time points, and a similar level was recorded for mice treated with 3.7 MBq. At day 8 after β-RIT, the Flt3-L concentration increased to 500 pg/mL in mice injected with 18.5 and 27.75 MBq of ^177^Lu-9E7.4 mAb. Levels returned to normal in both groups on around day 32 and 29, respectively, suggesting that myeloid toxicity was mild and reversible. Mice receiving a dose of 37 MBq exhibited a dramatic increase in Flt3-L level, reaching a mean of 1250 pg/mL, indicative of major myeloid toxicity that might account for the rapid mortality observed in that group.

**Figure 7:**
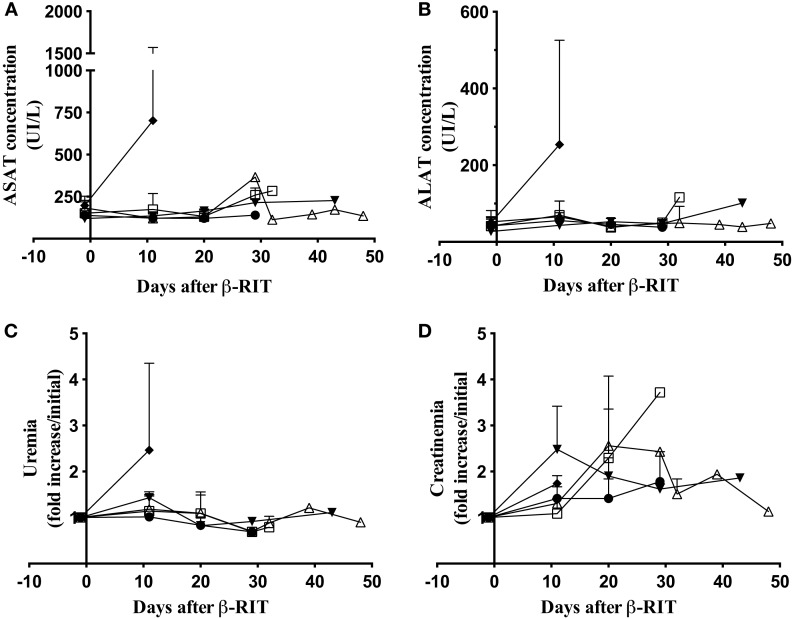
**Hepatic and kidney toxicity after β-RIT**. **(A)** ASAT, **(B)** ALAT, **(C)** urea, and **(D)** Creatinin concentrations were measured in pooled plasma from mice in the control group (●) or after β-RIT at 3.7 MBq (□), 18.5 MBq (△), 27.75 MBq (▼), or 37 MBq (♦). Results are expressed as mean ± SD. Data represent pooled plasma from three independent experiments.

We also assessed liver toxicity by monitoring level of ASAT and ALAT concentrations in plasma and kidney toxicity by measuring uremia and creatinemia. All markers were measured in the plasma at different time points after β-RIT (Figure 7). Figure 7A shows that, as in the control group, mice treated with 3.7, 18.5, and 27.75 MBq showed no variation in ASAT concentrations. In the 37 MBq group, a dramatic rise (≈700 UI/L) was noted 11 days after initiating the treatment. Similar results were observed for ALAT concentration (≈250 UI/L) and uremia (a 2.5-fold increase) (Figures 7B,C). Even though we noticed a certain variability in creatinemia over time in all the groups (Figure 7D), we observed a striking 2.5-fold increase in the 37 MBq group. Altogether, these results demonstrate that 37 MBq of ^177^Lu-9E7.4 mAb induces major liver and kidney toxicities.

### β-RIT with More Advanced Myeloma Disease

**Figure 8:**
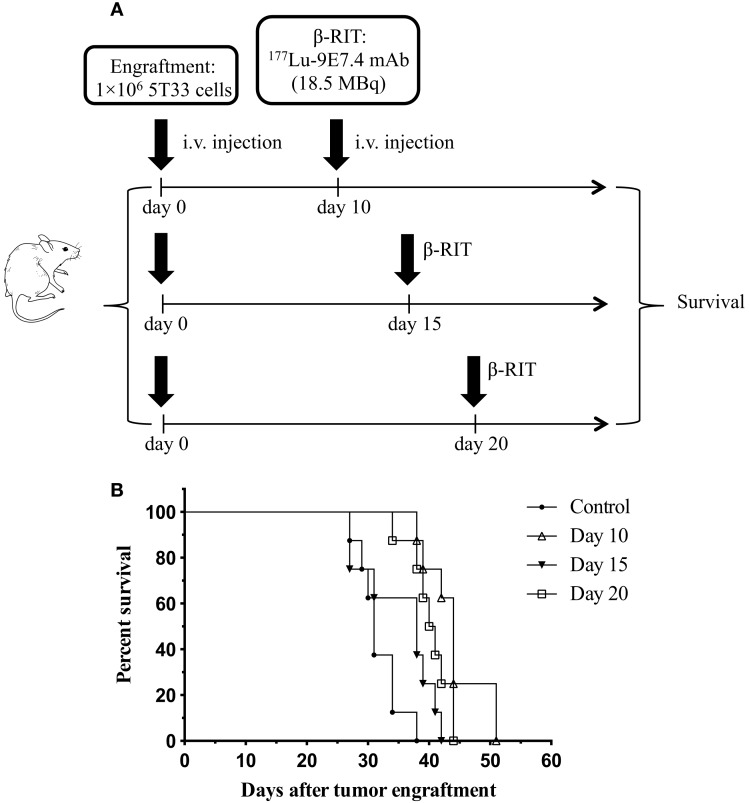
**Survival rates after treatment with β-RIT at different stages of MM development**. **(A)** At day 0, mice were engrafted with 1 × 10^6^ 5T33 cells. β-RIT (i.v. administration of 18.5 MBq ^177^Lu-9E7.4 mAb) was performed either at day 10, 15, or 20 after tumor engraftment; *n* = 8 mice per group. **(B)** Kaplan–Meier survival curves. The log-rank test indicated *p* = 0.0001 (day 10 vs. control) and *p* = 0.0004 (day 20 vs. control).

At the optimal activity of ^177^Lu-9E7.4 mAb, no cure and no major toxicity were observed in our study. We, therefore, hypothesized that β-RIT might be more efficient in the treatment of more advanced MM disease. Indeed, due to its physical properties, it has been shown that ^177^Lu is better adapted to the treatment of small solid tumors and metastases (11). We, thus, decided to test β-RIT at day 15 or 20 after tumor engraftment. Survival rates are shown in Figure 8. These data confirmed that survival was improved for mice treated at day 10 (44 days) compared to the control group (31 days). However, median survival for mice treated at day 15 or 20 after tumor engraftment did not improve further – 38 and 40.5 days, respectively. In addition, once again, no cure was recorded whatever the day of treatment, and none of the mice survived long term in any group. In summary, these results demonstrate that β-RIT is not an efficient treatment even for more advanced MM disease.

## Discussion

The literature describes promising results with RIT as a treatment for disseminated cancer or micrometastases. Most of the studies have been done in hematologic cancer and micrometastases [for review, see Ref. (11)]. MM is a malignant hemopathy mainly located in the bone marrow, and RIT has already been shown to be effective in treating human MM through targeting of CD138 (22). Our group has also demonstrated that α-RIT using the 281-2 anti-CD138 mAb is efficient in the treatment of MM in an immunocompetent mouse model (23). All these results suggested that β-RIT, targeting CD138 antigen on tumor cells, could also be a promising approach in this disease.

In this study, we developed a new anti-mouse CD138 mAb, 9E7.4, directed against a CD138-specific 40-aa peptide. Generation of proprietary hybridomas is an essential part of producing bispecific antibodies to reap the full benefit of these promising theranostic tools in nuclear medicine (40). The affinity of the 9E7.4 mAb for the CD138 antigen was similar to that of the 281-2 mAb, in the range of 10^−10^M. However, maximal binding of 9E7.4 mAb on CD138-expressing cells was at least twofold lower than maximal binding of 281-2 mAb (Figure 1). This difference might be due to the fact that they do not recognize the same epitope (data not shown), and how the two antibodies were generated. The 9E7.4 mAb was obtained using CD138-peptide immunization, while 281-2 was generated using CD138-expressing cells. Thus, the glycosylation status of the CD138 in the immunization could have been different, and this could influence binding of these antibodies (supported by our unpublished data).

With this new 9E7.4 anti-mouse CD138 mAb, we confirmed our previous results demonstrating that in the 5T33 immunocompetent MM mouse model, a single dose of α-RIT significantly raises median survival (80 vs. 37 days for control, untreated mice) and results in long-term survival for 45% of animals. In contrast, we showed that single-dose β-RIT performed in the same conditions was not sufficient to cure mice developing MM.

This lack of efficacy cannot be ascribed to the new anti-CD138 mAb since α-RIT was effective and tissue distributions of ^125^I-radioiodinated 281-2 and 9E7.4 mAbs were very similar. The only differences observed were a lower uptake in the liver and a higher uptake in the spleen with both ^125^I and ^177^Lu-radiolabeled 9E7.4 mAb compared to 281-2 mAb. These differences might be explained as mentioned above and will have to be further investigated. However, the difference in liver uptake could be correlated to the lower binding level of 9E7.4 mAb to CD138 compared to 281-2 mAb. Whatever the explanation, our results with ^177^Lu-9E7.4 mAb suggest that the therapeutic effect of β-RIT is not enough to offset its toxicity. Thus, accumulation of ^177^Lu-9E7.4 mAb appears to be deleterious in the bone marrow and liver. Many other studies performed with β-emitters have reported dose-limiting toxicity due to hematologic and bone marrow toxicities (19, 20, 41, 42). Here, we observed severe toxicity at 37 MBq at the hematologic, myeloid, hepatic, and kidney levels. Indeed, this activity was lethal and all the mice in this group had to be euthanized soon after RIT due to weight loss without any sign of disease progression (data not shown). A dramatic increase in Flt3-L in plasma confirmed that this treatment induced severe myeloid toxicity, as well as liver and kidney toxicity – as evidenced by ALAT, ASAT, urea, and creatinin plasma concentrations. At 27.75 MBq, overall toxicity was less pronounced, but hematologic toxicity remained significant with a dramatic but almost reversible nadir for leukocytes. In the group of mice treated with 18.5 MBq of ^177^Lu-9E7.4 mAb, the leukocyte count returned to normal more rapidly. Leukocytes are more sensitive than thrombocytes and erythrocytes because the main target of ionizing radiation is DNA, not present in either of the latter. Moreover, the absence of nucleus and their short life span (7–10 days), suggests that the drop in thrombocyte levels is probably due to destruction of their precursors in the bone marrow. This hypothesis is supported by the increase in plasma Flt3-L noted for these two groups. Based on the uremia, there was no apparent kidney toxicity in these two groups, although creatinemia indicated a certain level of toxicity at 27.75 MBq. However, this experiment was only performed once and will have to be confirmed. Finally, at these activity levels, no hepatic toxicity was observed, even though it is important to note that in the 27.75 MBq group, one mouse had to be sacrificed soon after treatment as it exhibited the same signs of toxicity as animals in the 37 MBq group. All these results combine to indicate that the potential efficacy of the treatment at 27.75 MBq is counterbalanced by its toxicity. This suggests that 18.5 MBq is not only the maximum tolerated dose, but also that this is the only activity, administered as a single dose, allowing a certain efficacy of the treatment. However, in the group which received 18.5 MBq, despite the lower toxicity and statistical improvement in median survival compared to the control group, no cure was effected. Mice in this group were euthanized because of paralysis and an increase in plasma paraprotein, with one exception (data not shown). Therefore, even if the treatment was associated with some toxicity, animals mainly died due to tumor burden.

^177^Lu emits β-ionizing radiation in a range of 2 mm within tissues, and the literature indicates that these β-particles are more efficient in hematologic cancers with a high-tumor burden (41). In our hands, 10 days after 5T33 MM engraftment, tumor development was weak, as previously shown using Luciferase-transfected 5T33 (23). Therefore, we thought that tumor burden at day 15 or 20 after tumor engraftment might be more appropriate for β-RIT treatment. However, even when β-RIT was performed on a more developed disease, no improvement in median survival was noted, and no cures were effected.

The difference between α-RIT and β-RIT in our MM model could be due to a difference in the radiosensitivity of 5T33 MM cells. Our group has already demonstrated that ^213^Bi-281-2 mAb killed the human MM cell line U266 more efficiently than ^131^I-281-2 mAb (26). Therefore, we will need to compare the sensitivity of 5T33 MM cells to α (^213^Bi) vs. β (^177^Lu) ionizing radiation at the cellular level.

Finally, although our study does not demonstrate any advantage of using β-RIT to treat MM, some therapeutic efficacy was observed with ^131^I-B-B4 mAb in a previous dosimetric study (22). In the previous study, two of the four patients treated exhibited either a stabilization of the paraprotein level for 1 year, or a break in its progression. In addition, the patient for whom paraprotein levels stabilized also reported an analgesic effect, with a decrease in disease-related bone pain, which made it possible for them to return to work. In these circumstances, we believe that β-RIT could improve therapeutic efficacy in this preclinical MM model, perhaps by using a fractionated dose strategy. Indeed, with a fractionation approach, the total activity administered can be higher than that delivered with a single dose, allowing improved efficacy compared to a single-dose regimen while reducing toxicity (43). Dose fractionation has already shown its efficacy, notably in treatment of NHL (44–46) and also for metastatic prostate cancer (47). Finally, Orozco et al. (48) demonstrated that β-RIT was less efficient when performed with ^177^Lu than with ^90^Y in an acute myeloid leukemia syngeneic mouse model. These authors hypothesized that the lower dose-rate and longer half-life of ^177^Lu could be responsible for the effect observed and suggested that dose fractionation is better adapted to the physical properties of this radionuclide. Therefore, further studies with the ^177^Lu-9E7.4 mAb in the immunocompetent MM mouse model will focus on assessing fractionated β-RIT treatment. In conclusion, this preclinical study indicates that, with a single dose, α-RIT is more efficient than β-RIT in treating MM in this mouse model.

## Author Contributions

Conception and design: NF, SG, CM, JB, LF, AM, FB, AF-C, JG, and MC. Method development: NF, SG, CM, JB, LF, AM, FB, AF-C, EB-C, FD, JG, and MC. Data acquisition: NF, SG, CM, EB-C, JG, and MC. Data analysis and interpretation: NF, SG, CM, JB, LF, AM, FB, AF-C, EB-C, FD, JG, and MC. Writing, review, and/or revision of the manuscript: NF, SG, JB, LF, AM, FB, AF-C, EB-C, FD, JG, and MC. Study supervision: JB, JG, and MC.

## Conflict of Interest Statement

The authors declare that the research was conducted in the absence of any commercial or financial relationships that could be construed as a potential conflict of interest.
