# Supplementary material for: Single-Dose Anti-CD138 Radioimmunotherapy: Bismuth-213 is More Efficient than Lutetium-177 for Treatment of Multiple Myeloma in a Preclinical Model
Source: Front Med (Lausanne). 2015 Nov 4;2:76. doi: 10.3389/fmed.2015.00076 (PMC4631990; doi:10.3389/fmed.2015.00076)
Supplement: Supplementary file 1 [file Image_1.PDF]

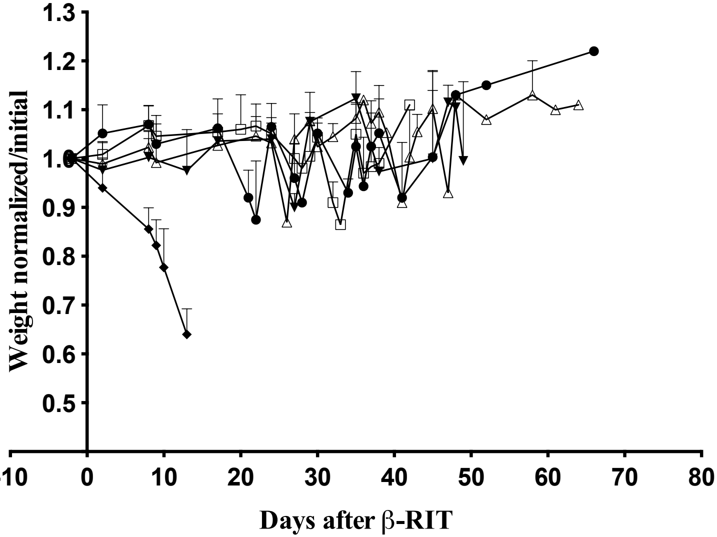

Supplementary figure 1. Mice weight monitoring after β-RIT. Mice, engrafted with $1{\times}10^6$ 5T33cells, were treated with different activities of ^{177}Lu-9E7.4 mAb at day 10: 3.7 MBq (□), 18.5 MBq (△), 27.75 MBq (▼), 37 MBq (◆) and control (●). Data represent three independent experiments with n = 8 (37 MBq), n = 11 (27.75 MBq), n = 14 (3.7 MBq), n = 15 (18.5 MBq) and n = 20 (control). Values are given as mean ± SD.
